# Insight into the research history and trends of total anomalous pulmonary venous connection: a bibliometric analysis

**DOI:** 10.1186/s13019-024-02787-8

**Published:** 2024-05-11

**Authors:** Chen Wen, Geng Shen, Chenhao Fang, Lan Tian

**Affiliations:** 1 Department of Cardiothoracic Surgery, Shanghai Children’s Medical Center, School of Medicine, Shanghai Jiao Tong University, Shanghai, China; 2 Division of Cardiology, Peking University First Hospital, Beijing, China; 3 Department of Neurosurgery, Shanghai Children’s Medical Center, School of Medicine, Shanghai Jiao Tong University, Shanghai, China; 4 Department of Pulmonary and Critical Care Medicine, Fujian Medical University Union Hospital, Fuzhou, China

**Keywords:** Bibliometrics, CiteSpace, VOSViewer, Total anomalous pulmonary venous connection, Hot spot, Research progress

## Abstract

**Background:**

Total anomalous pulmonary venous connection (TAPVC) is a rare congenital heart disease characterized by the inability of all pulmonary veins to connect to the left atrium. Our previous bibliometric article summarized the characteristics of only the 100 most cited papers in TAPVC research. The purpose of this study was to use comprehensive bibliometric analysis to examine the development history, current status, and future trends in the field of TAPVC.

**Methods:**

All publications on TAPVC published between 2000 and 2023 were collected from the Web of Science Core Collection. The publication and citation data were quantitatively analyzed by publication year, country, institution, author, and journal. Co-authorship and co-occurrence analyses were performed using VOSviewer, and keyword and reference bursts were identified using CiteSpace. Pearson’s test was used to examine the correlations between two continuous variables.

**Results:**

As of July 20, 2023, we identified 368 publications with 3320 citations. These publications were published in 132 journals and authored by 1835 researchers from 457 institutions in 47 countries. For the number of publications, the top country, top institution, top author, and top journals were the United States (*n* = 82), Shanghai Jiao Tong University (*n* = 13), Huiwen Chen (*n* = 9), and Annals of Thoracic Surgery and Pediatric Cardiology (*n* = 29 each), respectively. For the number of citations, the top country, top affiliation, top author, and top journal were the United States (*n* = 1348), University of Toronto (*n* = 250), Christopher A. Caldarone (*n* = 315), and Annals of Thoracic Surgery (*n* = 746), respectively. The number of national publications significantly correlated with GDP (*R* = 0.887, *P* < 0.001), research & development (R&D) expenditure (*R* = 0.375, *P* = 0.013), population (*R* = 0.694, *P* < 0.001), and journals (*R* = 0.751, *P* < 0.001). The number of national citations significantly correlated with GDP (*R* = 0.881, *P* < 0.001), R&D expenditure (*R* = 0.446, *P* = 0.003), population (*R* = 0.305, *P* = 0.037), and journals (*R* = 0.917, *P* < 0.001). International collaboration in the field of TAPVC was not well developed. The most commonly cited publication discussed era changes in mortality and reoperation rate in TAPVC patients. The most common keywords were “total anomalous pulmonary venous connection” and “congenital heart disease”. The keyword “case report” appeared most recently, with an average occurrence year of 2021.8. The co-occurrence analysis grouped 26 keywords into six themes: surgical repair of TAPVC, postoperative pulmonary vein stenosis, surgical repair of TAPVC patients with heterotaxy, application of echocardiography in diagnosing TAPVC, application of echocardiography in the prenatal diagnosis of TAPVC, and application of the sutureless technique in the surgical repair of TAPVC patients with right atrial isomerism or a single ventricle. Citation burst detection identified 32 references with citation bursts, seven of which had ongoing citation bursts until 2023.

**Conclusions:**

This study conducted a bibliometric analysis to provide a comprehensive overview of TAPVC research. We hope to offer new ideas for promoting development in the field of TAPVC.

**Supplementary Information:**

The online version contains supplementary material available at 10.1186/s13019-024-02787-8.

## Background

Total anomalous pulmonary venous connection (TAPVC) is a rare congenital anomaly that accounts for approximately 1–3% of congenital heart disease and is characterized by the inability of all pulmonary veins to connect to the left atrium [1]. The clinical presentation depends on the severity of pulmonary venous obstruction and the size of the right-to-left shunt. Patients usually present with symptoms in early childhood and are diagnosed within one year. When preoperative pulmonary venous obstruction is absent, pulmonary blood flow returns freely to the right atrium through the anomalous pulmonary connection. Subsequently, it crosses the foramen ovale, and the patient is usually born with mild symptoms. The pulmonary vascular resistance decreases after birth, excessive pulmonary circulation occurs, and elevated volume and pressure loads are transmitted to the right heart. Patients exhibit shortness of breath, tachycardia, and feeding difficulties and suffer from severe stunted growth, with most dying within the first year of life. If preoperative pulmonary venous obstruction is present, pulmonary venous hypertension and edema progress with increased pulmonary blood flow after birth. Hypoxemia and acidosis progress as a result of reflexive pulmonary artery vasoconstriction and poor gas exchange caused by pulmonary edema, and patients even develop symptoms similar to those of respiratory distress syndrome [2]. If left untreated, the mortality rate can reach 80% in the first year of life [3]. Surgery is the only effective treatment for TAPVC, and the underlying physiology of TAPVC determines the timing of surgery and the necessity of temporary measures. Elective surgical repair of TAPVC without preoperative pulmonary venous obstruction can be performed in neonates or postponed until 3–6 months of age. TAPVC with preoperative pulmonary venous obstruction is usually repaired by emergency surgery, which can be temporarily relieved through catheterization [4]. Although progress in diagnostic techniques, surgical interventions, and perioperative management has significantly improved the perioperative survival rate, postoperative pulmonary vein stenosis (PVS) remains a great challenge and leads to a disappointing prognosis [5]. Consequently, research on TAPVC is highly important. In recent years, TAPVC has received much attention, and there is an increasing body of publications on this topic. It is essential to understand the development history, current status, and future trends in the field of TAPVC.

In recent years, bibliometric analysis has become a common tool for analyzing the characteristics of a publication, such as publication year, countries, institutions, authors, journals, citations, and keywords. This analysis method allows researchers to determine the knowledge structure, development history, and future trends in a particular field [6]. With the increase in the number of scientific publications and the importance of research influence, bibliometric analysis plays an important role in evaluating research. Although bibliometric analysis has been widely used in the medical field, to our knowledge, only one bibliometric article on TAPVC research that we published earlier is available [7]. Our previous article only summarized the characteristics of the 100 most cited papers in the field of TAPVC. At present, there is no systematic or comprehensive bibliometric study on TAPVC. This new analysis explored the following specific aspects of TAPVC research. First, the number of publications and citations was examined by year, country, institution, author, journal, and keyword. Second, the correlations between a country’s number of publications and citations and its gross domestic product (GDP), percentage of GDP spent on research, population size, and number of journals were examined. Finally, keyword co-occurrence analysis was performed to understand the research themes. In general, a comprehensive analysis of the development history, research hotspots, and future trends in the field of TAPVC was performed using bibliometric tools. This study is expected to provide a reference and new ideas for promoting the development of TAPVC.

## Methods

### Data source

Compared with other databases such as Scopus, PubMed, and Medline, the Web of Science is more accurate and comprehensive and can serve as an important data source for medical researchers to perform bibliometric analysis [8]. All the relevant publications on TAPVC were retrieved using the advanced searching functionality of the Web of Science core collection (WoSCC) on July 20, 2023. The author developed the following search strategy after consulting with publication search specialists. The words “total anomalous pulmonary venous connection,” “total anomalous pulmonary venous drainage,” “total anomalous pulmonary venous return,” “TAPVC,” “TAPVD,” or “TAPVR” were used as search terms in the title. The publication year was limited to 2000–2023. Only publications written in English were included to promote analysis of the content. Because publication types such as meeting abstracts and editorials do not have the same frequency of citations and peer-review process, only publication types of articles or reviews were included in the analysis. After filtering, a full record with citations was downloaded from the database in plain text format. The impact factor (IF) of journals was obtained by manual retrieval through the Journal Citation Report (JCR) 2022 (Clarivate Analytics) [9]. The latest gross national product (GDP), research & development (R&D) expenditure, and population data for different countries are obtained from the World Bank.

### Bibliometric analysis

VOSviewer [10] and Citespace [11] are the main bibliometric analysis software. The number of accumulated publications was fitted with the publication year using SPSS software. The numbers of publications, citations, and keywords were analyzed using VOSviewer. Countries, institutions, authors, and journals were ranked by the number of publications or citations. A co-authorship relationship occurs when two authors participate in the same publication. Co-authorship analysis was used to reveal country, institution, and author collaborations. Collaborations between countries were visualized using an online bibliometric website (https://bibliometric.com). The frequency of collaboration between countries was calculated using the bibliometrix package in R [12]. The keywords represent the theme of the publication. Keyword co-occurrence refers to the existence of two different keywords in the same publication, and the keyword co-occurrence network can reveal research hotspots [13]. The emergence of keywords in different periods reflects the changing trend of research hotspots. A citation burst refers to a significant increase in the number of citations a publication receives, which lasts at least two years [11]. References and keywords that were associated with citation bursts were identified using CiteSpace. Pearson’s test was used to examine the correlations between the number of publications or citations and the GDP, R&D expenditure, population, and journals of different countries. The correlations between the number of publications or citations and the IFs of different journals were also investigated. A two-sided P value < 0.05 was considered to indicate statistical significance.

## Results

### Annual publication growth

**Fig. 1:**
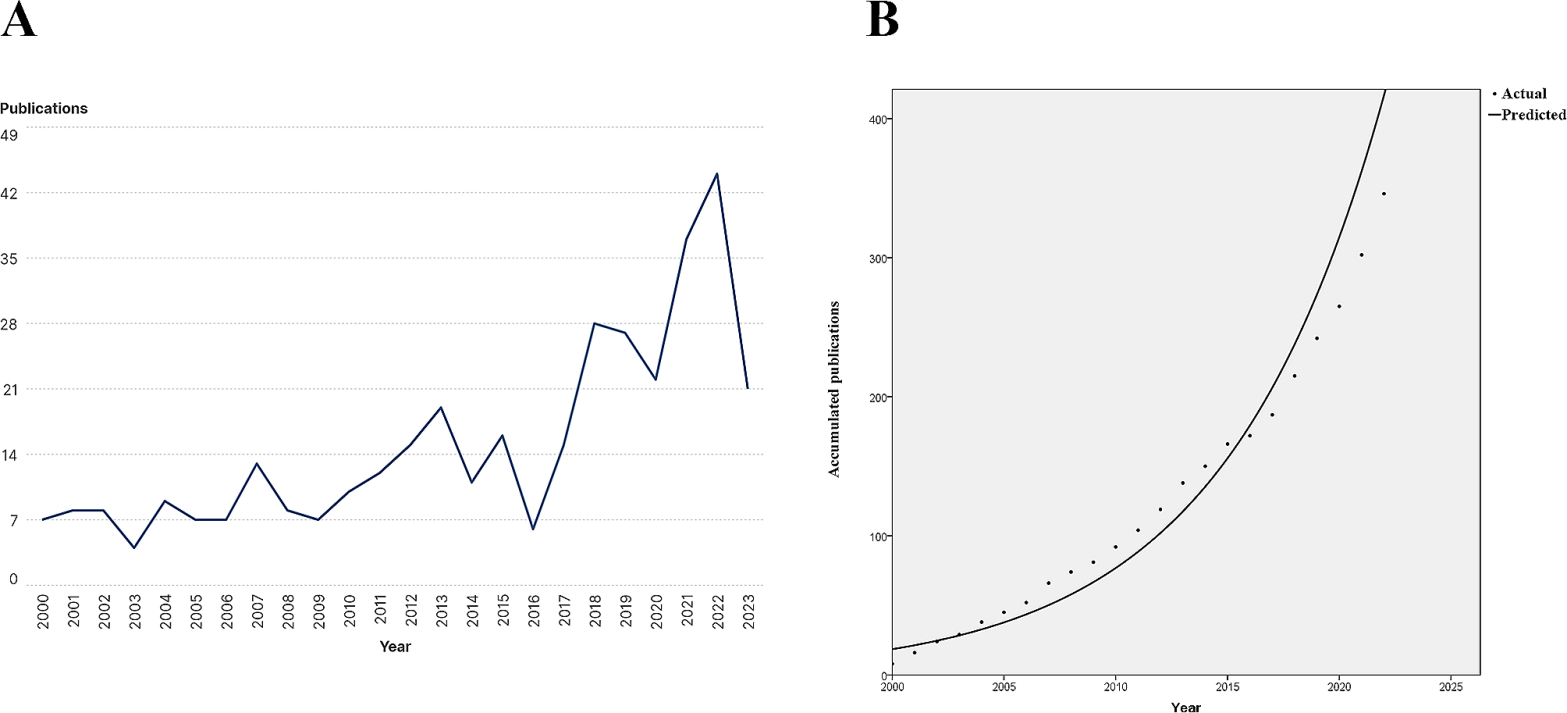
Number of publications per year **(A)** and the cumulative number of publications **(B)**

A total of 558 publications were retrieved, including 356 articles, 12 reviews, and 190 publications of other types. Articles and reviews (*n* = 368) were included in further analysis. The growth of publications with respect to publication year is shown in Fig. 1A. The number of publications on TAPVC showed an overall increasing trend, suggesting increasing interest in the research field of TAPVC. Before 2016, the number of publications fluctuated with the publication year. In subsequent years, the number of publications grew rapidly (the significant reduction in 2023 is attributed to incomplete data). An S curve function was utilized to explore the relationship between the cumulative number of publications and the publication year, which fit the trend well (R^2^ = 0.940) (Fig. 1B). This strong correlation suggests that the field of TAPVC is still in a period of rapid growth and development.

### National production of publications

**Fig. 2:**
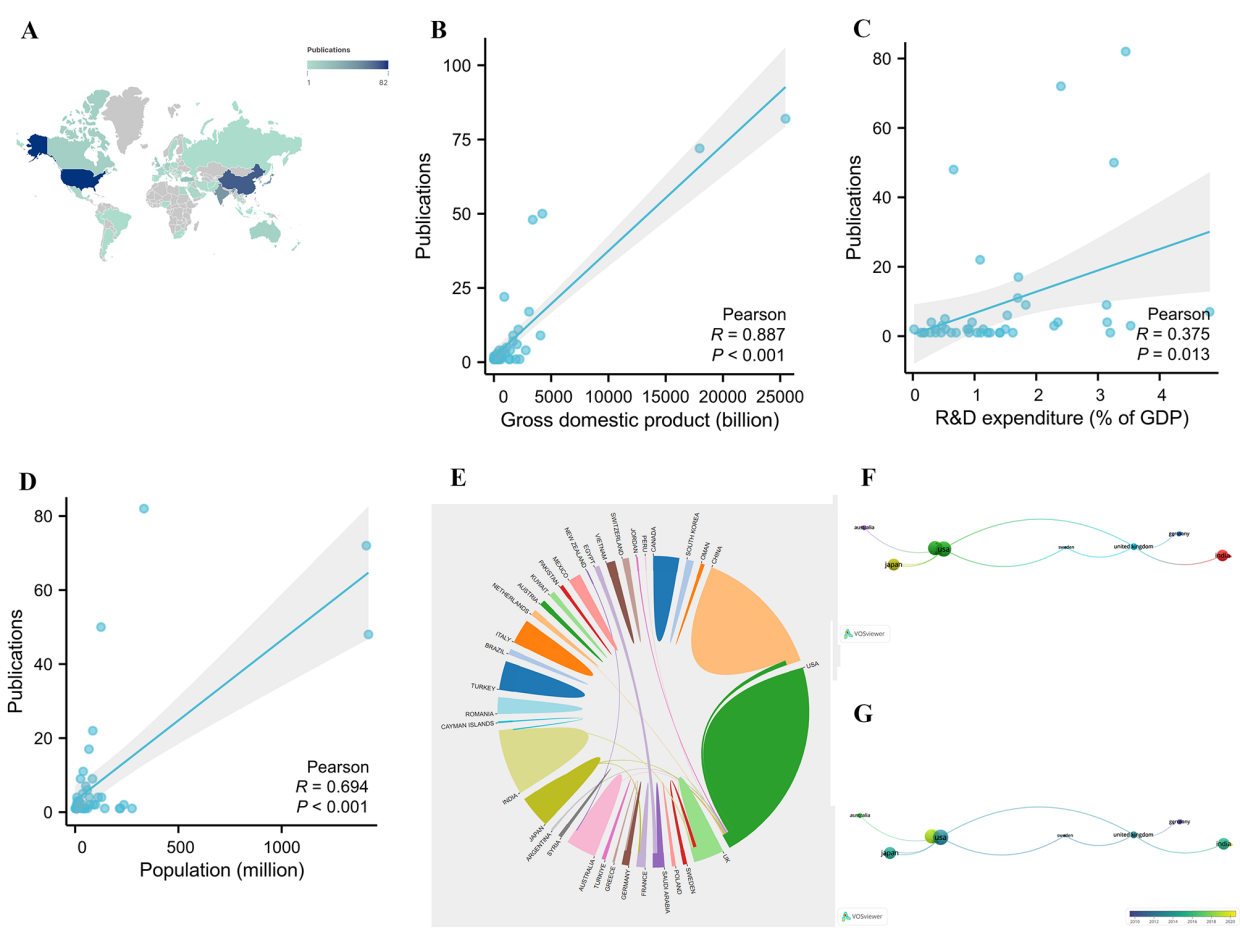
**(A)** Each country’s contribution to the field of total anomalous pulmonary venous connection; Pearson’s analysis revealed a significant correlation between the number of publications and gross domestic product **(B)**, research and development (R&D) expenditure **(C)** and population **(D)** of different countries. **(E)** Collaborations between countries; **(F)** Network clustering of country co-authorship analysis; **(G)** Time-overlapping network of country co-authorship analysis

To study each country’s contribution to the TAPVC field, the number of publications for each country was examined. A total of 47 countries/regions worldwide have conducted research on TAPVC (Fig. 2A). The top 20 countries/regions with the most publications are displayed in Supplementary Fig. 1, Additional File 1. There was no doubt that the United States was the main impetus in the field of TAPVC, with 82 publications, followed by China (*n* = 72), Japan (*n* = 50), and India (*n* = 48). Each of the remaining countries/regions has fewer 30 publications. Pearson’s analysis revealed that there was a significant correlation between the number of publications and the GDP (*R* = 0.887, *P* < 0.001) (Fig. 2B), R&D expenditure (*R* = 0.375, *P* = 0.013) (Fig. 2C) and population (*R* = 0.694, *P* < 0.001) (Fig. 2D) of different countries.

The collaborations between countries are displayed in Fig. 2E. The United States was the leader in international collaborations in the field of TAPVC. The most frequent collaborations were between the United States and the United Kingdom, the United States and China, the United States and Sweden, the United Kingdom and Sweden, the United Kingdom and India, and Egypt and Saudi Arabia, with a frequency of three each. To study the collaborations between countries, a co-authorship analysis of publications from these countries was performed (Fig. 2F). The circle size represents the number of publications, and the color represents the cluster. These 47 countries formed eight clusters, with the red and green clusters being the largest, each comprising four countries. The color of the circle in the time-overlapping network indicates the average year of publication for each country (Fig. 2G). Overall, there were very few connections between different clusters, indicating that international collaboration in the field of TAPVC needs to be better developed.

### Institutional production of publications

To study each institution’s contribution to the TAPVC field, the number of publications for each institution was examined. A total of 457 institutions worldwide were involved in the research field of TAPVC. The top 20 institutions with the most publications are shown in Supplementary Fig. 2, Additional File 1. Seven of the top 20 institutions were from China, followed by the United States and Australia, with 6 and 3 institutions, respectively. In terms of the specific institution, Shanghai Jiao Tong University topped the list with 13 publications, followed by All India Institute of Medical Sciences, Capital Medical University, and Children’s Hospital of Philadelphia with 9, 8, and 8 publications, respectively.

**Fig. 3:**
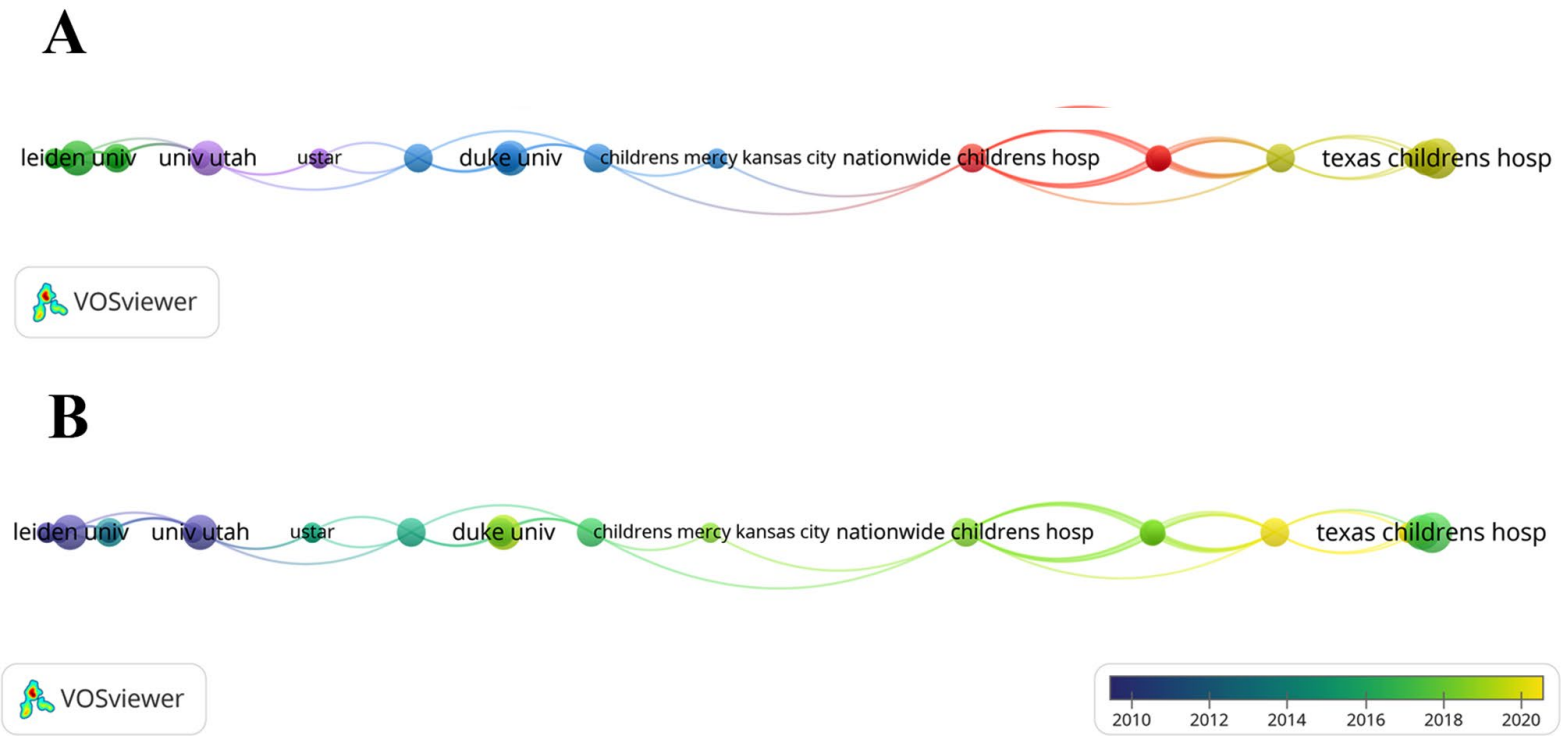
**(A)** Network clustering of institutional co-authorship analysis; **(B)** Time-overlapping network of institutional co-authorship analysis

To study the collaborations between institutions, a co-authorship analysis of publications from these institutions was performed (Fig. 3A). The circle size represents the number of publications, and the color represents the cluster. These 457 institutions formed five clusters, with the red cluster being the largest, which comprised 11 institutions, mainly from the United States. The time-overlapping network showed the average year of publication for each institution (Fig. 3B). Overall, there were very few connections between different clusters, indicating that institutional collaboration in the field of TAPVC was not well developed.

### Author production of publications

To study each author’s contribution to the TAPVC field, the number of publications for each institution was examined. A total of 1 835 authors have been committed to the field of TAPVC, and most (88.1%) have published only one publication. Each core author (2.8%) has published at least three publications, and these authors collaborated as research teams. The top 20 authors with the most publications are displayed in Supplementary Fig. 3, Additional File 1. Huiwen Chen was the greatest contributor, with 9 publications, followed by Guocheng Shi, with 8 publications. Christopher A. Caldarone, Yves d’Udekem, Igor E. Konstantinov, and Zhongqun Zhu each had 7 publications.

**Fig. 4:**
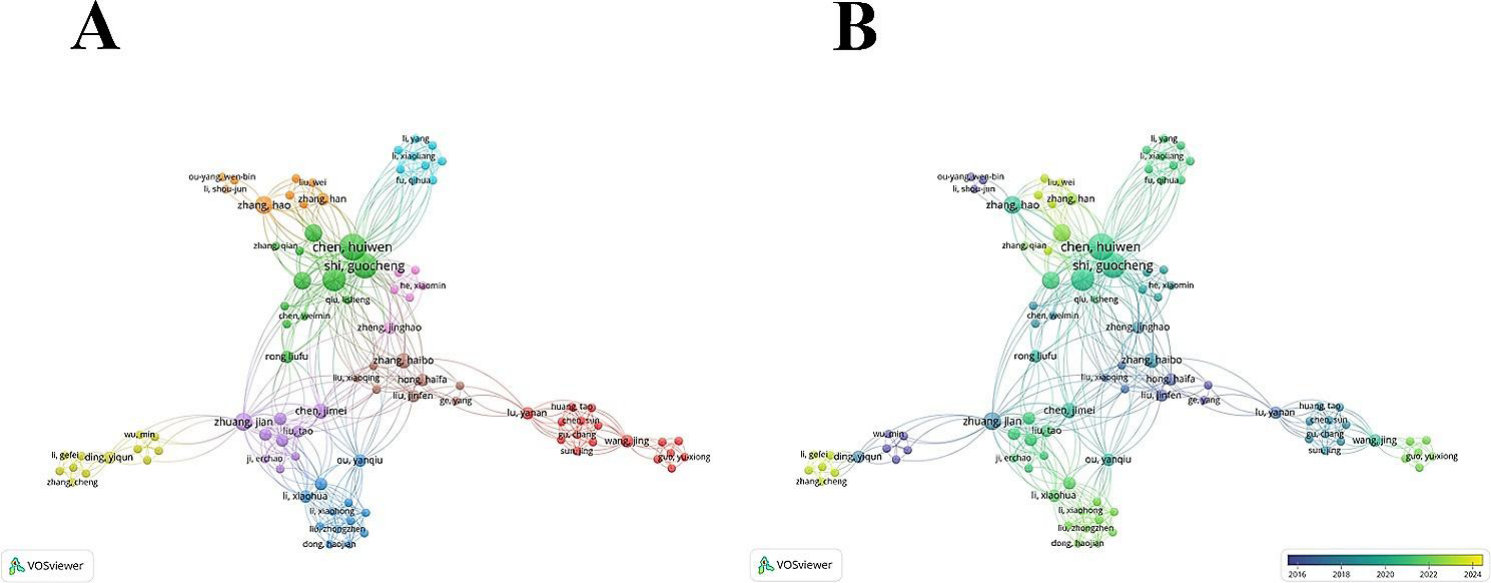
**(A)** Network clustering of the results of the researcher co-authorship analysis; **(B)** Time-overlapping network of the researcher co-authorship analysis

To study the collaboration between authors, a co-authorship analysis was performed, and the results are displayed in Fig. 4A. The circle size represents the number of publications, and the color represents the cluster. These authors formed 9 clusters, with the red cluster being the largest, comprising 17 authors, mainly from Shanghai Jiao Tong University. The time-overlapping network showed the average year of publication for each author (Fig. 4B). Researchers from China have formed research networks in the field of TAPVC.

### Disciplinary distribution of publications

To study the disciplinary distribution in the field of TAPVC, the number of publications for each WOS category was examined. The four disciplines with the most publications were cardiac & cardiovascular systems (*n* = 244), surgery (*n* = 120), pediatrics (*n* = 81), and respiratory system (*n* = 72). Other disciplines included radiology, nuclear medicine & medical imaging (*n* = 28), medicine, general & internal (*n* = 24), obstetrics & gynecology (*n* = 15), acoustics (*n* = 15), genetics & heredity (*n* = 11), and peripheral vascular disease (*n* = 9). This indicated that the research performed in this field was broad and diverse and could attract the attention of researchers from different fields.

### Journal production of publications

**Fig. 5:**
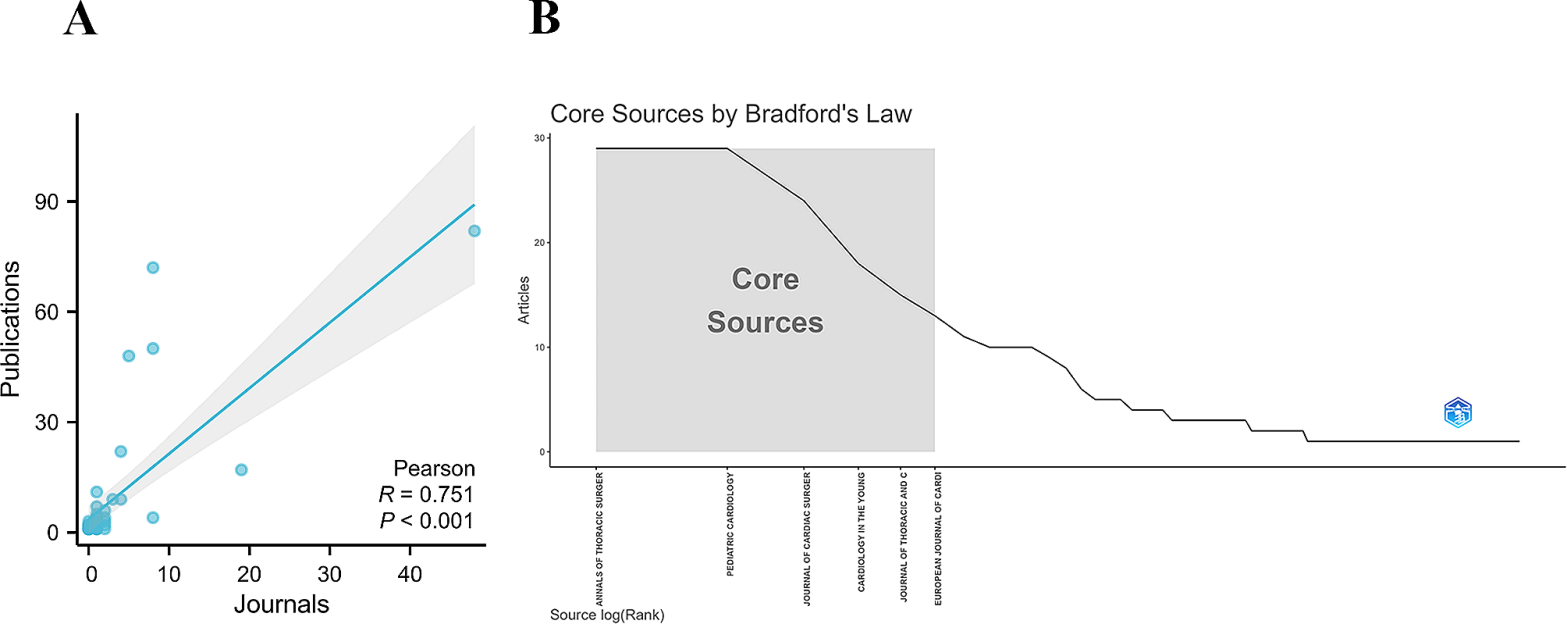
**(A)** Pearson’s analysis revealed a significant correlation between the number of publications and the number of journals in different countries; **(B)** Bradford’s law showed that six core journals published 128 publications

These 368 publications were published in 132 journals. The top 20 journals with the most publications are displayed in Supplementary Table 1, Additional File 1. Annals of Thoracic Surgery and Pediatric Cardiology topped the list with 29 publications each, followed by Journal of Cardiac Surgery with 24 publications. Each of the remaining journals had fewer than 20 publications. There were 48 journals from the United States, followed by the United Kingdom with 19 journals. China, Japan, and Switzerland each had eight journals. Pearson’s analysis revealed a significant correlation between the number of publications and the number of journals (*R* = 0.751, *P* < 0.001) in different countries (Fig. 5A). Bradford’s law showed that six core journals published 128 publications (Fig. 5B). These findings can aid researchers in selecting the most appropriate journals to publish their manuscripts.

### Most cited publications

Citation count is one of the indices used to judge the academic influence of a publication. Highly cited publications usually represent important research topics in a specific field. To study the influence of publications in the field of TAPVC, the number of citations each publication received was examined. The 20 most cited publications are displayed in Supplementary Table 2, Additional File 1, and all of them have been cited more than 40 times. Notably, nineteen of the 20 most-cited publications were published in JCR Q1 journals, and the remaining one was in JCR Q2 journal, indicating that these publications were published in high-level journals. Of these 20 publications, 15 focused on surgical treatment, two examined the application of echocardiography in prenatal diagnosis, two investigated the pathogenic mechanism of TAPVC, and the remaining one examined late neurodevelopmental problems after surgical repair of TAPVC. A publication published in Circulation in 2007 has received the most citations: “Factors associated with mortality and reoperation in 377 children with total anomalous pulmonary venous connection” [14]. This study involved a large single-institution cohort of patients whose mortality after surgical repair of TAPVC decreased but remained high in young patients and those with cardiac TAPVC or preoperative PVS. Despite improvements in perioperative care, unfavorable anatomic characteristics remain important risk factors for postoperative mortality. The second most cited publication was also published in Circulation: “Total anomalous pulmonary venous connection: morphology and outcome from an international population-based study” [15]. This international population-based study investigated the morphological characteristics of TAPVC and explored risk factors for postoperative mortality and PVS. The third most cited publication was published in Annals of Thoracic Surgery in 2005: “Total anomalous pulmonary venous connection: An analysis of current management strategies in a single institution” [16]. This study explored the impact of management strategies on postoperative mortality and PVS and revealed that the prognosis of patients with a single ventricle was still worse than that of patients with two ventricles.

### National citations

**Fig. 6:**
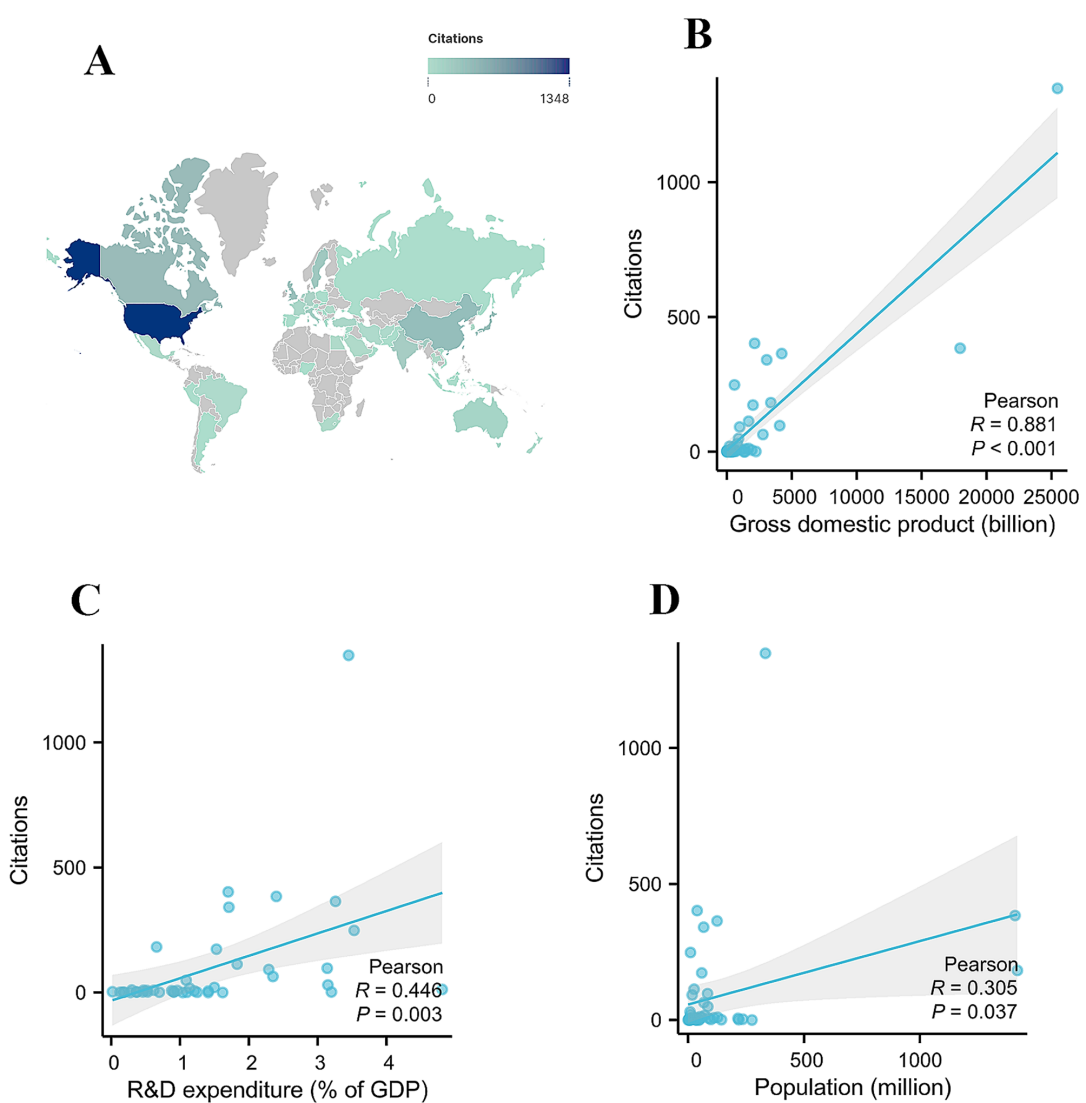
**(A)** The number of citations each country received in the field of total anomalous pulmonary venous connection; Pearson’s analysis revealed that there was a significant correlation between the number of citations and gross domestic product **(B)**, research and development (R&D) expenditure **(C)**, and population **(D)** of different countries

To study the influence of each country in the field of TAPVC, the number of citations each country received was examined (Fig. 6A). The top 20 countries/regions with the most citations are displayed in Supplementary Fig. 4, Additional File 1. The United States ranked first (*n* = 1348), followed by Canada (*n* = 402). China (*n* = 384), Japan (*n* = 364), and the United Kingdom (*n* = 341). Each of the remaining countries had fewer than 300 citations. Pearson’s analysis revealed that there was a significant correlation between the number of citations and the GDP (*R* = 0.881, *P* < 0.001) (Fig. 6B), R&D expenditure (*R* = 0.446, *P* = 0.003) (Fig. 6C), and population (*R* = 0.305, *P* = 0.037) (Fig. 6D) of different countries.

### Institutional citations

To study the influence of each institution in the field of TAPVC, the number of citations each institution received was examined. The top 20 institutions with the most citations are shown in Supplementary Fig. 5, Additional File 1. Six of the top 20 institutions were from the United States, followed by the United Kingdom, which has four institutions. In terms of the specific institution, the University of Toronto topped the list with 250 citations, followed by Queen Silvia Children’s Hospital, University of London Imperial College of Science, Technology and Medicine, and Children’s Hospital of Philadelphia with 248, 248 and 233 citations, respectively. Each of the remaining institutions had fewer than 200 citations.

### Author citations

To study the influence of each author in the field of TAPVC, the number of citations each author received was examined. The top 20 authors with the most citations are displayed in Supplementary Fig. 6, Additional File 1. Christopher A. Caldarone topped the list with 315 citations, followed by John G. Coles and Glen S. Van Arsdell with 271 citations each. Each of the remaining authors had fewer than 200 citations.

### Journal citations

**Fig. 7:**
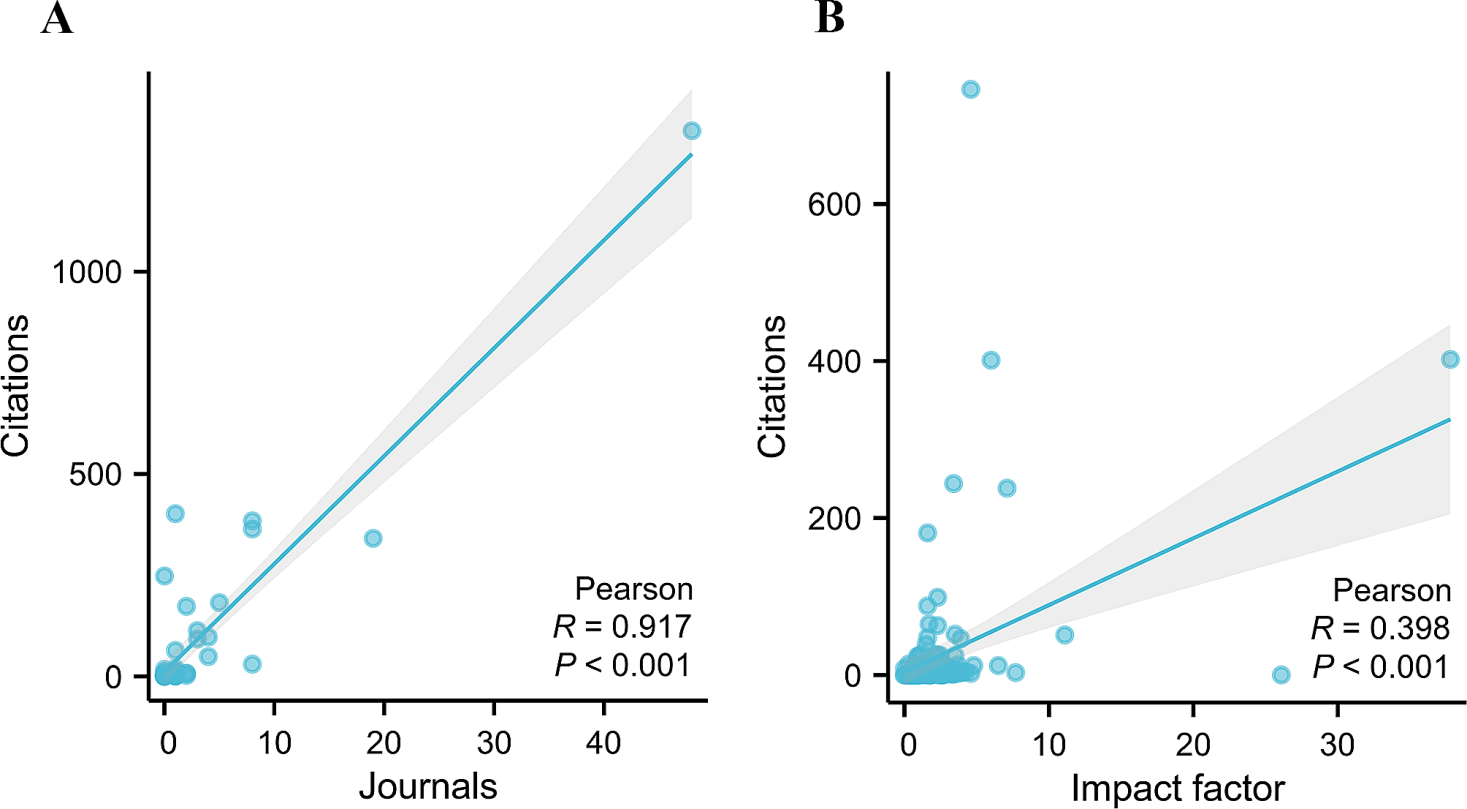
**(A)** Pearson’s analysis revealed a significant correlation between the number of citations and the number of journals of different countries; **(B)**There was a significant correlation between the number of citations and the impact factor of different journals

To study the influence of each journal in the field of TAPVC, the number of citations each journal received was examined. The top 20 journals with the most publications are displayed in Supplementary Table 3, Additional File 1. Annals of Thoracic Surgery topped the list with 746 citations, followed by Circulation and Journal of Thoracic and Cardiovascular Surgery with 402 and 401 citations, respectively. Each of the remaining journals had fewer than 300 citations. Pearson’s analysis revealed a significant correlation between the number of citations and the number of journals (*R* = 0.917, *P* < 0.001) in different countries (Fig. 7A). There was also a significant correlation between the number of citations and the IF of different journals (*R* = 0.398, *P* < 0.001) (Fig. 7B).

### Citation bursts of references

**Fig. 8:**
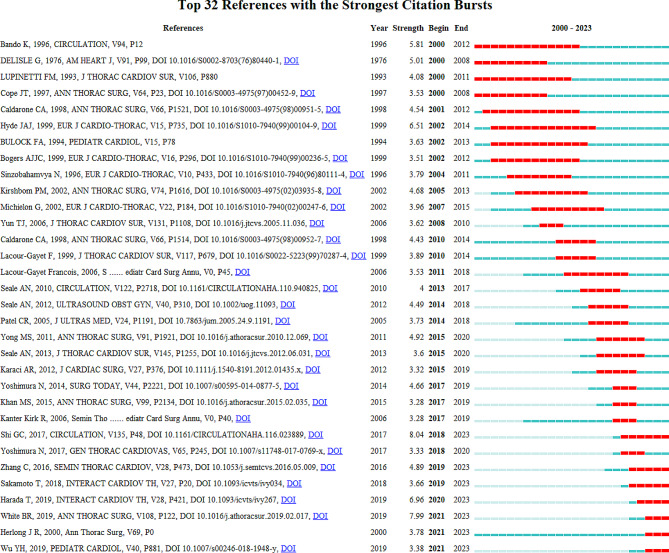
Citation burst detection identified 32 references with citation bursts

Citation burst detection was performed to capture the rapid increase in the popularity of references over a specific period. A total of 32 references with citation bursts from 2000 to 2023 were identified (Fig. 8). The blue line represents the time interval from 2000 to 2023, and the red line represents the period of citation bursts. The publication “Total Anomalous Pulmonary Venous Connection: The Current Management Strategies in a Pediatric Cohort of 768 Patients”, published in Circulation, had the highest citation burst value of 8.04 [5]. This multicenter study employed a large cohort of patients (768 individuals) to investigate the influence of current therapeutic regimens on the results of TAPVC. This study revealed that surgical treatment in patients with TAPVC could yield an acceptable outcome and investigated risk factors associated with mortality and postoperative PVS. This study explored the effects of the sutureless technique and revealed that this technique decreased the incidence of postoperative PVS in patients with preoperative PVS but not in patients without preoperative PVS. The publication “Repair of Total Anomalous Pulmonary Venous Connection: Risk Factors for Postoperative Obstruction” published in Annals of Thoracic Surgery, had the second highest citation burst value of 7.99 [17]. Because the definition of preoperative PVS has widely varied in prior studies, this research graded the severity of preoperative PVS to precisely examine the risk factors for postoperative PVS. This study may help in the risk stratification of patients with TAPVC. The publication “Surgical results of total anomalous pulmonary venous connection repair in 256 patients”, published in Interactive Cardiovascular and Thoracic Surgery, had the third highest citation burst value of 6.96 [18]. This study revealed that the long-term outcomes of surgical repair for TAPVC patients were satisfactory, and predictors of mortality and postoperative PVS were investigated. Postoperative PVS was strongly related to mortality, and close follow-up was needed, particularly within six months after surgery. The most recent burst occurred in 2021 and has lasted for three years. Seven references with ongoing citation bursts until 2023 deserve our further attention. Of these publications, six focused on the surgical treatment of TAPVC, and the remaining study focused on the classification of pulmonary venous malformations. One meta-analysis published by Wu et al. is worth noting [19]. This meta-analysis included 26 studies involving a total of 2702 patients and compared outcomes between the sutureless technique and conventional repair and revealed that the sutureless technique could reduce the incidence of postoperative PVS and re-operations, but could not reduce postoperative early, late, or overall mortality. The popularity of these research topics is likely to continue, and they could thus become potential frontiers in the future.

### Frequency of keywords

Keywords were the high condensation and summary of the main contents of a publication, which could indicate the research theme. Frequency analysis of keywords can help researchers quickly identify the main research hotspots in a research field. Supplementary Fig. 7, Additional File 1 shows the top 20 keywords with the greatest frequency. There was no doubt that “total anomalous pulmonary venous connection” topped the lists with a frequency of 181, followed by “congenital heart disease” (*n* = 60), “pulmonary vein stenosis” (*n* = 41), “echocardiography” (*n* = 20), “pulmonary vein” (*n* = 20), “sutureless technique” (*n* = 16), “prenatal diagnosis” (*n* = 13), “surgery” (*n* = 13), “fetal echocardiography” (*n* = 12), and “single ventricle” (*n* = 11).

### Major research areas

**Fig. 9:**
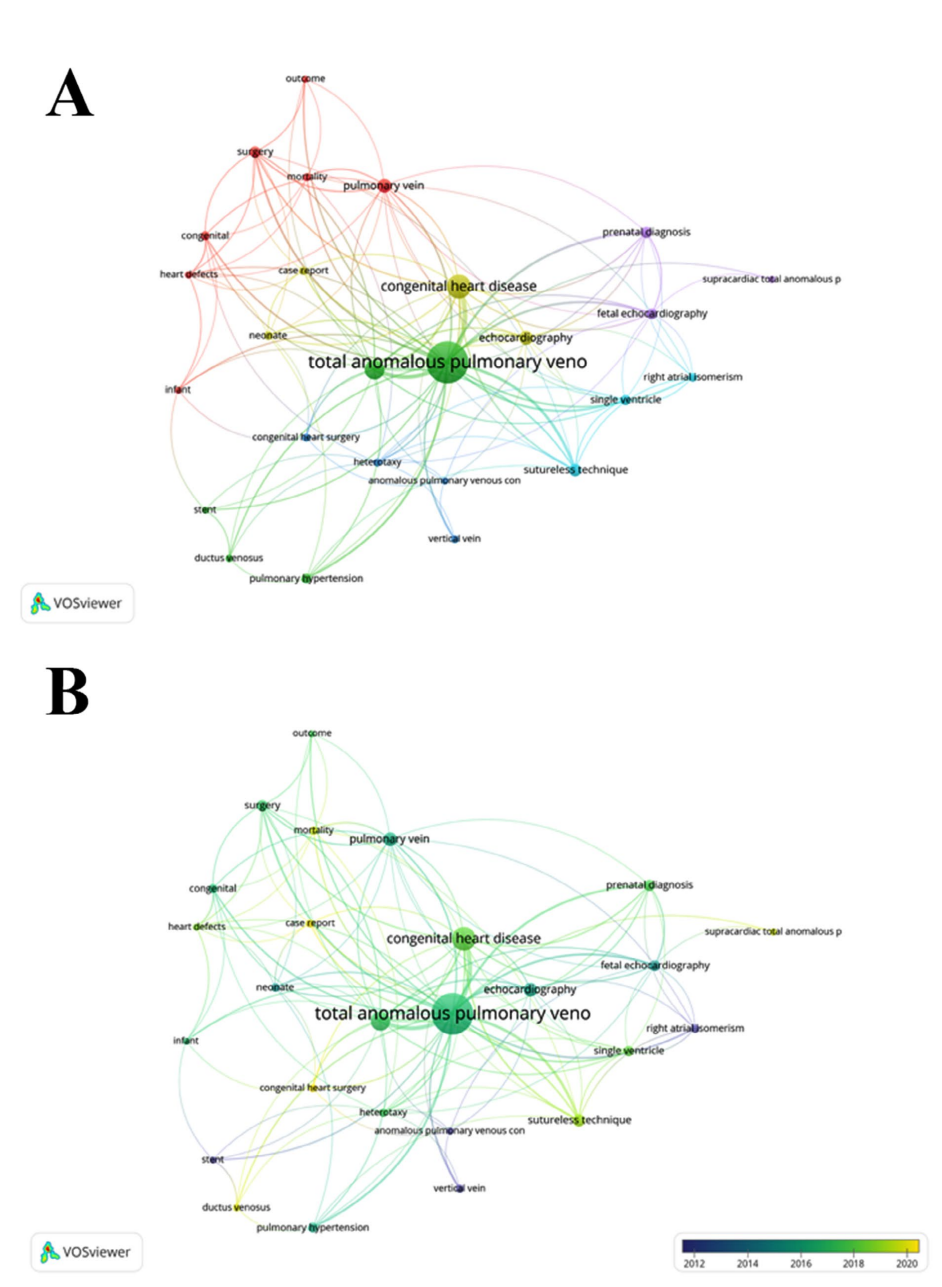
**(A)** Network clustering of keyword co-occurrence analysis; **(B)** Time-overlapping co-occurrence network of keywords

Keyword co-occurrence analysis is an important approach for investigating the main research interests and hot issues in a specific research field. Among 546 keywords, 35 met the threshold of 5 occurrences. If keywords had the same meanings, they were merged. After merging duplicate keywords, 26 (threshold setting of 5) were displayed in the network visualization (Fig. 9A). The node size represents the frequency of the keywords, and the distance between them represents the strength of the association. Closely related keywords were grouped into one cluster, and each cluster represented one research domain. The 26 keywords were grouped into six clusters. The red cluster was the largest and included the keywords “congenital,” “heart defects,” “infant,” “mortality,” “outcome,” “pulmonary vein,” and “surgery” This indicates that the most important topic was surgical repair of TAPVC. The green cluster included the keywords such as “total anomalous pulmonary venous connection,” “pulmonary vein stenosis,” “pulmonary hypertension,” and “stent” and was mainly associated with postoperative PVS, an important complication after surgical repair of TAPVC. The blue cluster included the keywords such as “anomalous pulmonary venous connection,” “congenital heart surgery,” and “heterotaxy” and was mainly related to the surgical repair of TAPVC patients with heterotaxy. The yellow cluster included the keywords such as “congenital heart disease” and “echocardiography” and was mainly related to the application of echocardiography in diagnosing TAPVC. The purple cluster included the keywords such as “fetal echocardiography” and “prenatal diagnosis” and was mainly related to the application of echocardiography in the prenatal diagnosis of TAPVC. Finally, the light blue cluster included the keywords “right atrial isomerism,” “single ventricle,” and “sutureless technique” and was mainly related to the application of the sutureless technique in the surgical repair of TAPVC patients with right atrial isomerism or a single ventricle.

Figure 9B shows the visualization of the time overlapping of keywords. Different nodes are marked with different colors based on the average occurrence year of these keywords. The keyword “case report” appeared most recently, with an average occurrence year of 2021.8.

### Keyword bursts

Citation burst detection was conducted to explore the rapid increase in popularity of keywords over a specific period. In this study, there were no keywords with citation bursts. This suggests that research topics are relatively smooth in the field of TAPVC.

## Discussion

This bibliometric study quantitatively analyzed the characteristics of publications in the field of TAPVC and comprehensively reviewed its development history, current status, and future trends. The present study is important for several reasons. Scholars can be aware of research hotspots and set future study directions. This bibliometric study can serve as an educational tool for residents to familiarize themselves with TAPVC development. Journal editors can recognize potentially important manuscripts based on prior knowledge. Clinicians can apply the latest findings to clinical practice to achieve better results. The government may formulate future funding policies and research investment plans.

Research growth in the field of TAPVC has experienced two stages according to the increasing number of publications over time. Before 2016, there was a long, slow growth stage of knowledge accumulation, and it was essential to have practical clinical experience rather than technical improvement. In 2017, a publication published in Circulation used a large patient cohort to examine the current outcome of TAPVC patients [5]. Since 2017, TAPVC research has entered a rapid growth stage, indicating that it has entered a phase of rapid development. During the second stage, the importance of this medical condition is recognized, researchers have become interested in this topic, and research outputs in this field have substantially increased. Overall, the rapid growth trend in TAPVC research will likely continue. Despite the fast-growing trend of research outputs in the field of TAPVC, studies on TAPVC are generally still lagging behind those on other cardiovascular diseases, such as hypertension and arteriosclerosis. The rarity of this disease is to blame for the lower research activity in the field of TAPVC compared with more mature fields.

The United States is in a dominant position in terms of quantity and quality and is the core of international collaboration in the field of TAPVC. The United States is dominant in many other fields, indicating that the United States is at the cutting edge in the scientific community. The present study showed that greater number of journals, a greater GDP, a greater population, and greater R&D expenditure promote research productivity and impact in the field of TAPVC. GDP represents the total value of all productive activities over a time period and is the primary measure of a country’s strength. It is widely believed that the economic conditions of a country will affect its research output. The current study’s results align with previous research showing a link between GDP and scientific research output [20]. Policy-makers in less developed countries need to provide more support to maintain the growth trend of knowledge in the field of TAPVC. Only in this way could medical staff perform better clinical interventions and thus improve outcomes in clinical practice with the support of these strong-evidence studies [20]. The investment in R&D determines the level of infrastructure and resources for research, the contribution to scientific progress and innovation, and the creation of new knowledge [21]. Countries with limited research output should increase the proportion of R&D investment to improve scientific productivity and impact. Finding this correlation is particularly important in the current economic climate. The financial pressure on the government has contributed to a reduction in research funding. A direct correlation between research investment and scientific output could demonstrate the benefits of continued support for research funding. University and university hospitals are the birthplaces of medical knowledge. Countries with low research output should increase the number of universities and hospitals. Consistent with previous research, our study shows that countries with more indexed journals produce more scientific work [21]. Researchers tend to submit manuscripts to journals from their own countries and cite journals from their own countries. Countries with low research output should establish more internationally influential journals. The population is an important factor in TAPVC research. TAPVC is extremely rare; only an adequate population can accumulate enough cases. The extremely low incidence of TAPVC suggests that a sufficient number of patients can be included in studies based only on a sufficient population. A national medical center with centralized resources and knowledge should be established to collect cases scattered throughout the country. International collaboration and the sharing and exchange of patient data should be encouraged to increase the number of patients worldwide for research. In addition, registries and databases should be established to centralize dispersed patients [22]. When interpreting these results, however, some confounding factors need to be taken into account. Because domestic papers are published faster, it is possible that researchers from some countries tend to publish their findings in local journals. Such publications are not included in the present study. Only recently have some countries published papers in international journals. In addition, a previous study showed that proficiency in English facilitated the publication of articles in international journals [23]. Manuscripts from English-speaking countries have a greater chance of being accepted than those from non-native English-speaking countries.

Inadequate international research collaboration is a potential reason for the limited research outputs in the field of TAPVC. International collaboration is one of the future development directions. TAPVC is extremely rare, and a limited number of patients and researchers may hinder TAPVC research in many countries. International research collaboration will increase research productivity and impact in this field [24]. Collaborative works enhance the sharing of researchers and patients among countries to produce more research outcomes. The lack of international collaboration is difficult to explain but may be due to a lack of funding, communications, favorable circumstances, or international conferences in this field. Nonetheless, the included publications are limited to those published in English, which can distort the actual situation of international collaboration. To improve collaboration, we need to form societies on pediatric cardiac surgery, hold international and regional academic conferences, establish specialized journals on congenital heart surgery, create international databases on congenital heart surgery, provide global management for patients with congenital heart disease, strengthen education, research and community service, promote international exchange of trainees, provide educational programs for medical staff coming from different countries, facilitate international teaching and treatment, certify congenital heart surgeons globally to move surgeons to areas of need, promote international collaboration by contacting leading authors or research groups at the global level, encourage collaboration in intergovernmental organizations, give aid loans, donate equipment to hospitals, provide services and public infrastructures, offer training scholarships for foreign doctors, establish new pediatric cardiac surgery centres, and sponsor overseas medical missions [25, 26].

The journal analysis showed that Annals of Thoracic Surgery had the largest number of publications and citations in this field of TAPVC. This journal has an IF of 4.6 (2022) and focuses on cardiothoracic surgery. Researchers tend to publish and cite publications in this journal because this journal is a highly ranked academic journal in the field of cardiothoracic surgery, and high-ranking academic journals are always looking for novel and valuable research. Bradford’s law showed that six core journals published 128 publications. The main idea of Bradford’s law is that core journals publish the most articles that are widely cited [27]. When researchers stray from core journals, the impact of published articles declines. This trend has resulted in the majority of highly cited papers coming from a small number of professional journals. There was a significant correlation between the number of citations and the IF of different journals. Our research obeys the well-known rule that most cited articles are published in journals with high IFs, which in turn leads to the high IFs of these journals.

Co-occurrence analysis of keywords identified six themes: surgical repair of TAPVC, postoperative PVS, surgical repair of TAPVC patients with heterotaxy, application of echocardiography in diagnosing TAPVC, application of echocardiography in the prenatal diagnosis of TAPVC, and application of sutureless technique in the surgical repair of TAPVC patients with right atrial isomerism or a single ventricle.

Without intervention, the mortality rate during the first year of life is as high as 80%. Surgery is the only effective treatment for this disease. Due to improved diagnostic accuracy, surgical approaches, and perioperative management, early mortality is steadily decreasing, but there is still considerable variability. The early mortality rate reported in recent publications ranges between < 10% and 20%. Surgical management of TAPVC remains a challenge [28].

PVS is the most common complication after surgical repair of TAPVC with an incidence of 10–20%, and remains an issue. PVS is associated with high morbidity and mortality following surgical repair. PVS usually occurs within a few months after surgical repair and progresses rapidly. The effective methods for preventing postoperative PVS are still unknown and are worth exploring [28].

Heterotaxy syndrome is frequently associated with TAPVC. The outcomes of patients with heterotaxy syndrome who undergo TAPVC repair remain unclear, and there are limited data. Some studies have reported that heterotaxy syndrome is a significant risk factor, while others have found no significant difference in the survival of heterotaxy patients [29].

The surgical results of TAPVC depend on a detailed understanding of the anatomical morphology. Echocardiography remains the first-line diagnostic modality for TAPVC because of its safety and accessibility. However, echocardiography has disadvantages, such as poor spatial resolution, an acoustic window, and poor interpretation accuracy. Echocardiography exhibits good performance in diagnosing isolated TAPVC but has limited accuracy in diagnosing mixed or infracardiac TAPVC and TAPVC associated with obstruction, heterotaxy syndrome, or right atrial isomerism. Improving the diagnostic accuracy of echocardiography in challenging cases remains to be addressed [30].

Prenatal diagnosis of TAPVC, especially obstructive TAPVC, is essential for birth planning and postpartum management and decreases morbidity and mortality in the perinatal period. Fetal heart echocardiography is the primary diagnostic modality. However, the prenatal diagnosis of TAPVC is very challenging, and misdiagnosis and missed diagnosis are extremely common [31].

Single ventricle and right atrial isomerism are frequently associated with TAPVC. In such patients, abnormal localization of thoracic structures, such as the great arteries and systemic veins, results in compression of pulmonary venous drainage. The outcomes of patients with a single ventricle or right atrial isomerism who undergo surgical repair of TAPVC remain poor and are associated with a high risk of postoperative PVS and mortality [32, 33]. The effective treatment for these patients remains elusive. The sutureless technique has been introduced to reduce the risk of postoperative PVS. In this technique, the pulmonary venous confluence and the left atrium were opened, and the left atrium wall was sutured to the mediastinal pleura. The sutureless technique was favored because of its broad applicability to different anatomical morphologies and avoidance of direct anastomosis of the pulmonary vein endothelium. The efficacy, applicability, and indications of the sutureless technique are still not well established [34]. The favorable results of the sutureless technique in some recent studies have extended the indications of this technique to difficult patients, such as those with a single ventricle or right atrial isomerism [33]. However, the outcomes of the sutureless technique in such patients remain uncertain.

There are some limitations to this present bibliometric analysis. First, the search strategy was performed only in the WoSCC database. Because the WoSCC database includes most high -quality publications, it does not distort the overall trend of the research results. Second, this study included only publications written in English and omitted those written in native languages. Second, recently published high-quality studies may not have received sufficient attention due to citation delays and need to be updated in subsequent studies. Third, more citations do not always mean higher quality and controversial publications tend to have more citations. Fourth, only articles and reviews were included. However, these two types of publications are more likely to represent contributions to a research field. Finally, some limitations inherent in bibliometrics should be considered. For example, the importance of recently published publications is underestimated because there is not enough time to accumulate citations.

## Conclusions

This bibliometric analysis identified the most influential publications and identified the most prominent countries, institutions, authors, and journals in the field of TAPVC. The field of TAPVC is still in a period of rapid growth and development. A greater number of journals, GDP, population, and research & development expenditure promote research productivity and impact. International collaboration in the field of TAPVC should be encouraged. The research themes in this field are focused on six areas: surgical repair of TAPVC, postoperative pulmonary vein stenosis, surgical repair of TAPVC patients with heterotaxy, application of echocardiography in diagnosing TAPVC, application of echocardiography in the prenatal diagnosis of TAPVC, and application of the sutureless technique in the surgical repair of TAPVC patients with right atrial isomerism or a single ventricle. This study is the first bibliometric analysis to provide a comprehensive overview of TAPVC research. Our findings could provide valuable information on the development history, current status, and future trends, as well as new ideas for promoting development in this field.

### Electronic supplementary material

Below is the link to the electronic supplementary material.

Supplementary Material 1

## Data Availability

The datasets used and/or analysed during the current study are available from the corresponding author on reasonable request.

## Abbreviations

TAPVC: Total anomalous pulmonary venous connection
PVS: Pulmonary vein stenosis
WoSCC: Web of Science core collection
IF: Impact factor
JCR: Journal Citation Report
GDP: Gross national product
R&D: Research & development
