# Supplementary material for: Insight into the research history and trends of total anomalous pulmonary venous connection: a bibliometric analysis
Source: J Cardiothorac Surg. 2024 May 11;19:285. doi: 10.1186/s13019-024-02787-8 (PMC11088122; doi:10.1186/s13019-024-02787-8)

**SUPPLEMENTAL MATERIAL**

**Supplementary Table 1** Top 20 journals with the most publications in the field of total anomalous pulmonary venous connection

| **Rank** | **Journal** | **Publications** | **Country** | **Impact factor** | **JCR-c** |
| --- | --- | --- | --- | --- | --- |
| 1 | Annals of Thoracic Surgery | 29 | USA | 4.6 | Q1 |
| 2 | Pediatric Cardiology | 29 | USA | 1.6 | Q3 |
| 3 | Journal of Cardiac Surgery | 24 | USA | 1.6 | Q3 |
| 4 | Cardiology in the Young | 18 | USA | 1 | Q3 |
| 5 | Journal of Thoracic and Cardiovascular Surgery | 15 | USA | 6 | Q1 |
| 6 | European Journal of Cardio-Thoracic Surgery | 13 | Netherlands | 3.4 | Q1 |
| 7 | Journal of Cardiothoracic Surgery | 11 | United Kingdom | 1.6 | Q3 |
| 8 | Annals of Pediatric Cardiology | 10 | USA | 0.7 | Q4 |
| 9 | Catheterization and Cardiovascular Interventions | 10 | USA | 2.3 | Q3 |
| 10 | World Journal for Pediatric and Congenital Heart Surgery | 10 | USA | 0.9 | Q4 |
| 11 | Ultrasound in Obstetrics & Gynecology | 9 | USA | 7.1 | Q1 |
| 12 | Frontiers in Cardiovascular Medicine | 8 | Switzerland | 3.6 | Q2 |
| 13 | Interactive Cardiovascular and Thoracic Surgery | 6 | United Kingdom | 2 | Q3 |
| 14 | Heart Surgery Forum | 5 | USA | 0.6 | Q4 |
| 15 | Pediatric Radiology | 5 | USA | 2.3 | Q2 |
| 16 | Progress in Pediatric Cardiology | 5 | USA | 0.9 | Q4 |
| 17 | Echocardiography-A Journal of Cardiovascular Ultrasound and Allied Techniques | 4 | USA | 1.5 | Q4 |
| 18 | Journal of The American Society Of Echocardiography | 4 | USA | 6.5 | Q1 |
| 19 | Journal of Ultrasound in Medicine | 4 | USA | 2.3 | Q2 |
| 20 | Turkish Journal of Thoracic and Cardiovascular Surgery | 4 | Turkey | 0.6 | Q4 |

**Supplementary Table 2** Top 20 publications with the most citations in the field of total anomalous pulmonary venous connection

| **Rank** | **Title** | **First author** | **Publication year** | **Journal** | **JCR** | **Total citations** | **TC per Year** |
| --- | --- | --- | --- | --- | --- | --- | --- |
| 1 | Factors associated with mortality and reoperation in 377 children with total anomalous pulmonary venous connection | Tara Karamlou | 2007 | Circulation | Q1 | 166 | 9.76 |
| 2 | Total Anomalous Pulmonary Venous Connection Morphology and Outcome From an International Population-Based Study | Anna N. Seale | 2010 | Circulation | Q1 | 145 | 10.36 |
| 3 | Total anomalous pulmonary venous connection: An analysis of current management strategies in a single institution | Camille L. Hancock Friesen | 2005 | Annals of Thoracic Surgery | Q1 | 140 | 7.37 |
| 4 | Total Anomalous Pulmonary Venous Connection The Current Management Strategies in a Pediatric Cohort of 768 Patients | Guocheng Shi | 2017 | Circulation | Q1 | 91 | 13.00 |
| 5 | Total anomalous pulmonary venous connection: Results of surgical repair of 100 patients at a single institution | Angela M. Kelle | 2010 | Journal of Thoracic and Cardiovascular Surgery | Q1 | 85 | 6.07 |
| 6 | Partial and total anomalous pulmonary venous connection in the fetus: two-dimensional and Doppler echocardiographic findings | E. R. Valsangiacomo | 2003 | Ultrasound In Obstetrics & Gynecology | Q1 | 74 | 3.52 |
| 7 | Management of pulmonary venous obstruction after correction of TAPVC: risk factors for adverse outcome | M. Ricci | 2003 | European Journal of Cardio-Thoracic Surgery | Q1 | 68 | 3.24 |
| 8 | Preoperative pulmonary venous obstruction affects long-term outcome for survivors of total anomalous pulmonary venous connection repair | Paul M. Kirshbom | 2002 | Annals of Thoracic Surgery | Q1 | 61 | 2.77 |
| 9 | Late neurodevelopmental outcome after repair of total anomalous pulmonary venous connection | Paul M. Kirshbom | 2005 | Journal of Thoracic and Cardiovascular Surgery | Q1 | 59 | 3.11 |
| 10 | Outcomes of Surgery for Simple Total Anomalous Pulmonary Venous Drainage in Neonates | Matthew S. Yong | 2011 | Annals of Thoracic Surgery | Q1 | 58 | 4.46 |
| 11 | Total anomalous pulmonary venous connection: long-term appraisal with evolving technical solutions | Guido Michielon | 2002 | European Journal of Cardio-Thoracic Surgery | Q1 | 57 | 2.59 |
| 12 | Primary sutureless repair for "simple" total anomalous pulmonary venous connection: Midterm results in a single institution | Bobby Yanagawa | 2011 | Journal of Thoracic and Cardiovascular Surgery | Q1 | 56 | 4.31 |
| 13 | Heterotaxy patients with total anomalous pulmonary venous return: Improving surgical results | David L. S. Morales | 2006 | Annals of Thoracic Surgery | Q1 | 53 | 2.94 |
| 14 | Total anomalous pulmonary venous connection: impact of prenatal diagnosis | A. N. Seale | 2012 | Ultrasound In Obstetrics & Gynecology | Q1 | 52 | 4.33 |
| 15 | Dysregulation of the PDGFRA gene causes inflow tract anomalies including TAPVR: integrating evidence from human genetics and model organisms | Steven B. Bleyl | 2010 | Human Molecular Genetics | Q2 | 52 | 3.71 |
| 16 | Total anomalous pulmonary venous connection: Outcome of postoperative pulmonary venous obstruction | Anna N. Seale | 2013 | Journal of Thoracic and Cardiovascular Surgery | Q1 | 51 | 4.64 |
| 17 | Next-generation sequencing identifies novel genes with rare variants in total anomalous pulmonary venous connection | Xin Shi | 2018 | Ebiomedicine | Q1 | 51 | 8.50 |
| 18 | Total Anomalous Pulmonary Venous Connection: Factors Associated With Mortality and Recurrent Pulmonary Venous Obstruction | S. Adil Husain | 2012 | Annals of Thoracic Surgery | Q1 | 50 | 4.17 |
| 19 | Primary Sutureless Repair for Infants With Mixed Total Anomalous Pulmonary Venous Drainage | Osami Honjo | 2010 | Annals of Thoracic Surgery | Q1 | 49 | 3.50 |
| 20 | Improving outcomes in functional single ventricle and total anomalous pulmonary venous connection | Andrew J. Lodge | 2004 | Annals of Thoracic Surgery | Q1 | 47 | 2.35 |

**Supplementary Table 3** Top 20 journals with the most citations in the field of total anomalous pulmonary venous connection

| **Rank** | **Journal** | **Citations** | **Country** | **Impact factor** | **JCR-c** |
| --- | --- | --- | --- | --- | --- |
| 1 | Annals of Thoracic Surgery | 746 | USA | 4.6 | Q1 |
| 2 | Circulation | 402 | USA | 37.8 | Q1 |
| 3 | Journal of Thoracic and Cardiovascular Surgery | 401 | USA | 6 | Q1 |
| 4 | European Journal of Cardio-Thoracic Surgery | 244 | Netherlands | 3.4 | Q1 |
| 5 | Ultrasound in Obstetrics & Gynecology | 238 | USA | 7.1 | Q1 |
| 6 | Pediatric Cardiology | 181 | USA | 1.6 | Q3 |
| 7 | Catheterization and Cardiovascular Interventions | 99 | USA | 2.3 | Q3 |
| 8 | Journal of Cardiac Surgery | 88 | USA | 1.6 | Q3 |
| 9 | Interactive Cardiovascular and Thoracic Surgery | 65 | United Kingdom | 2 | Q3 |
| 10 | Journal of Ultrasound in Medicine | 63 | USA | 2.3 | Q2 |
| 11 | Human Molecular Genetics | 52 | United Kingdom | 3.5 | Q2 |
| 12 | Ebiomedicine | 51 | United Kingdom | 11.1 | Q1 |
| 13 | Journal of Cardiothoracic Surgery | 48 | United Kingdom | 1.6 | Q3 |
| 14 | Human Mutation | 46 | USA | 3.9 | Q2 |
| 15 | Thoracic and Cardiovascular Surgeon | 39 | Germany | 1.5 | Q3 |
| 16 | General Thoracic and Cardiovascular Surgery | 27 | Japan | 1.2 | Q3 |
| 17 | Birth Defects Research | 26 | USA | 2.1 | Q3 |
| 18 | Pediatric Radiology | 26 | USA | 2.3 | Q2 |
| 19 | International Journal of Cardiology | 25 | Ireland | 3.5 | Q2 |
| 20 | Surgery Today | 25 | Japan | 2.5 | Q2 |

**Supplementary Fig. 1** The top 20 countries with the most publications


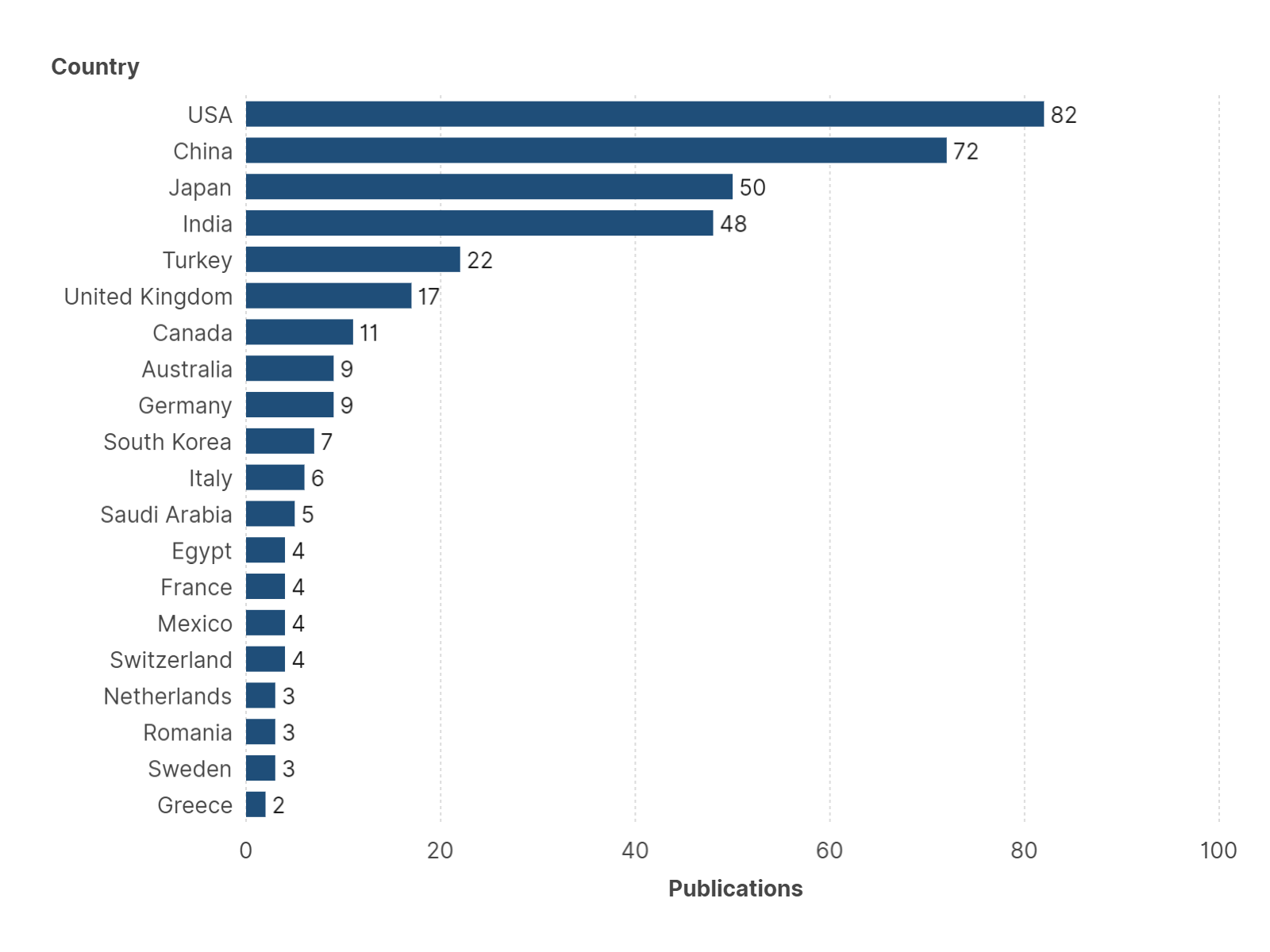


**Supplementary Fig. 2** The top 20 institutions with the most publications in the field of total anomalous pulmonary venous connection


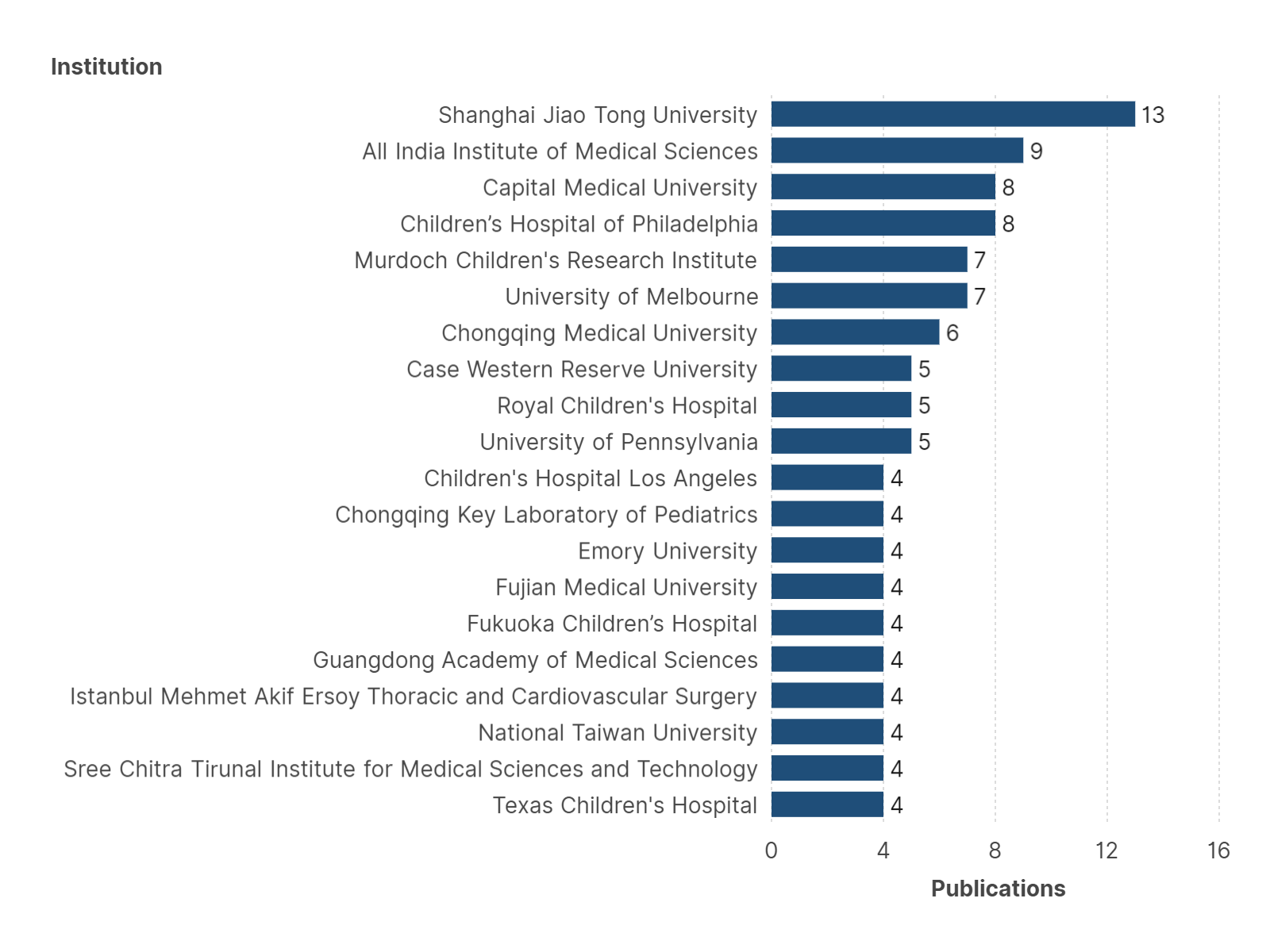


**Supplementary Fig. 3** The top 20 authors with the most publications in the field of total anomalous pulmonary venous connection


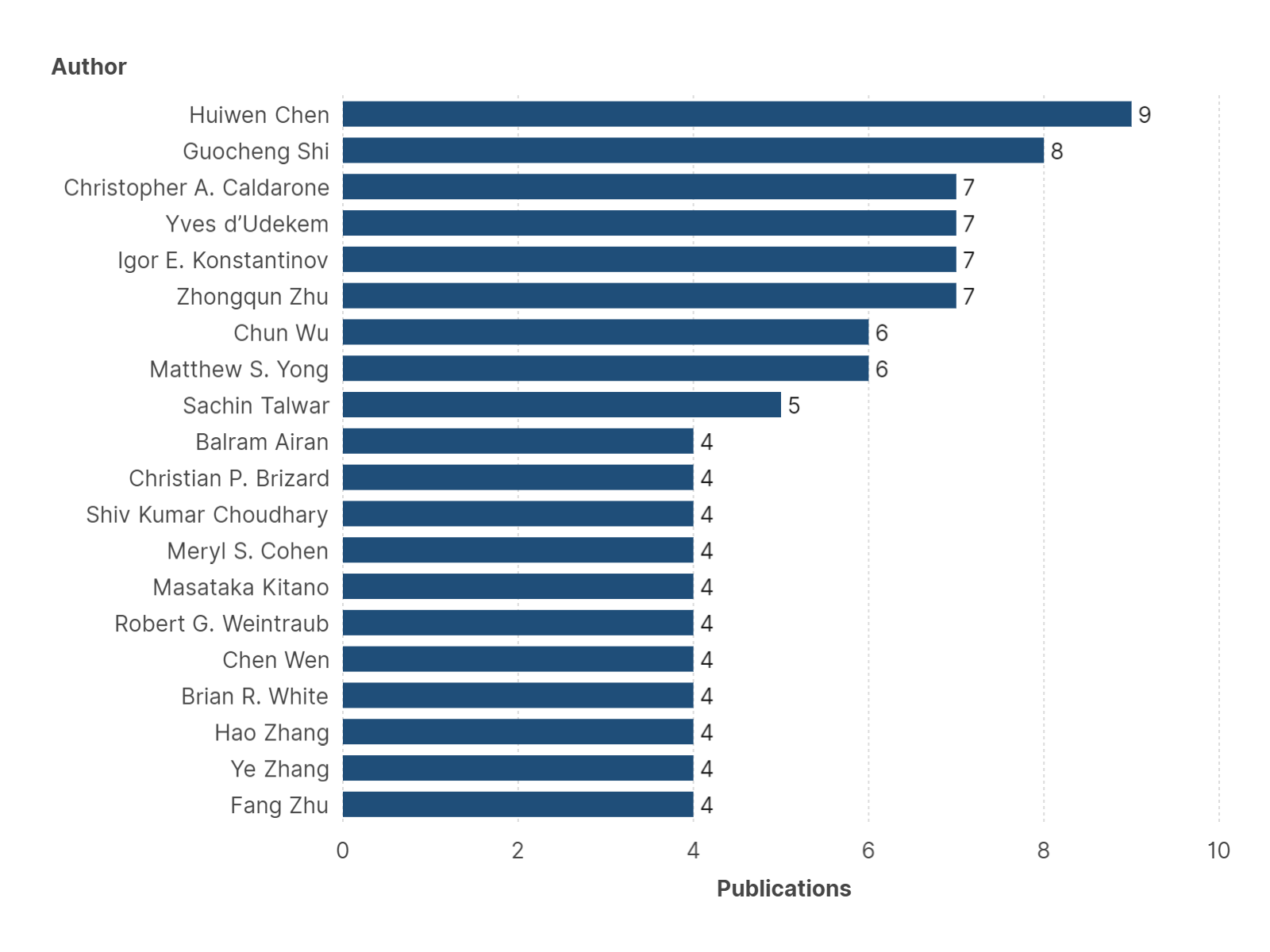


**Supplementary Fig. 4** The top 20 countries with the most citations in the field of total anomalous pulmonary venous connection


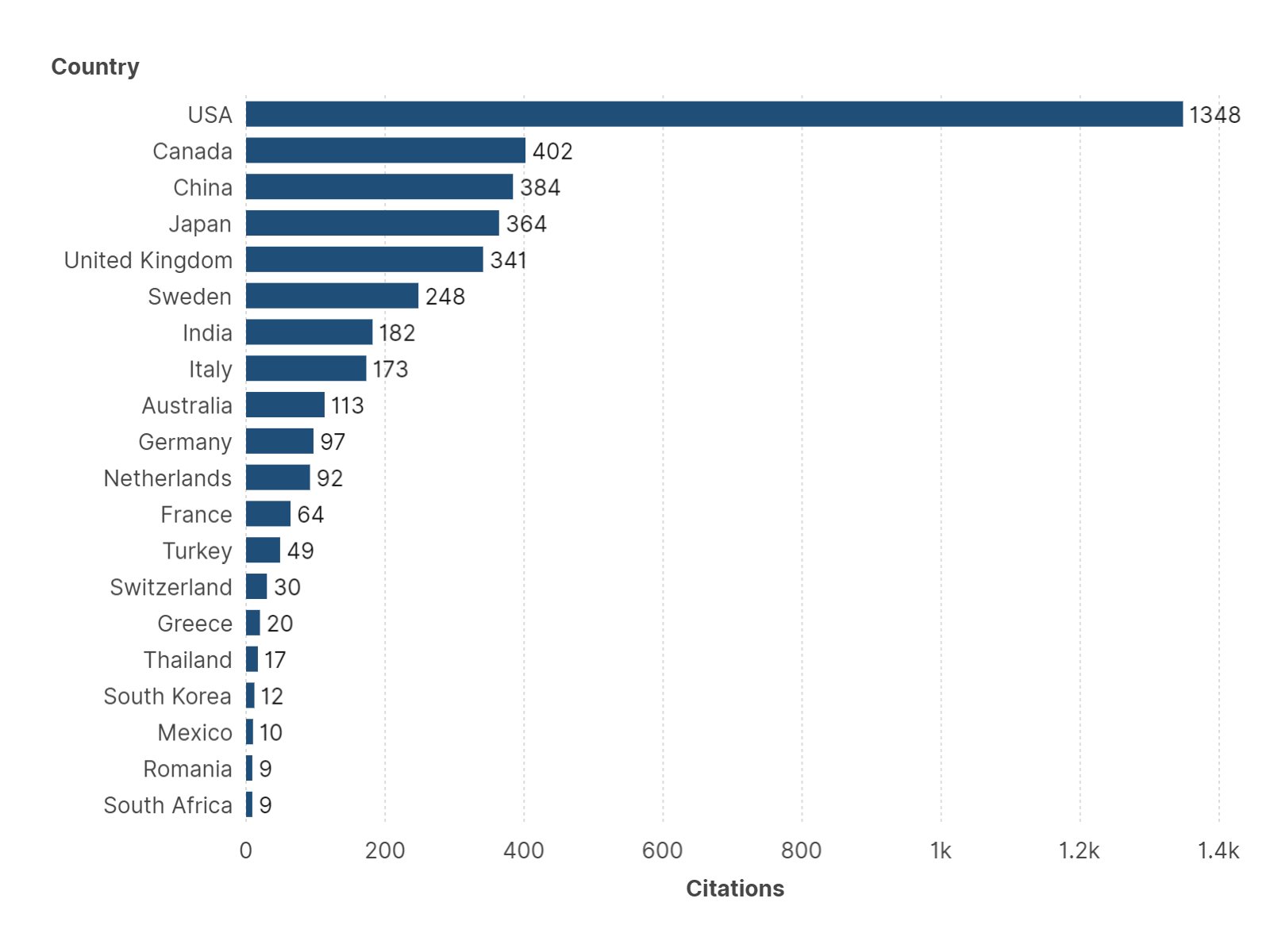


**Supplementary Fig. 5** The top 20 institutions with the most citations in the field of total anomalous pulmonary venous connection


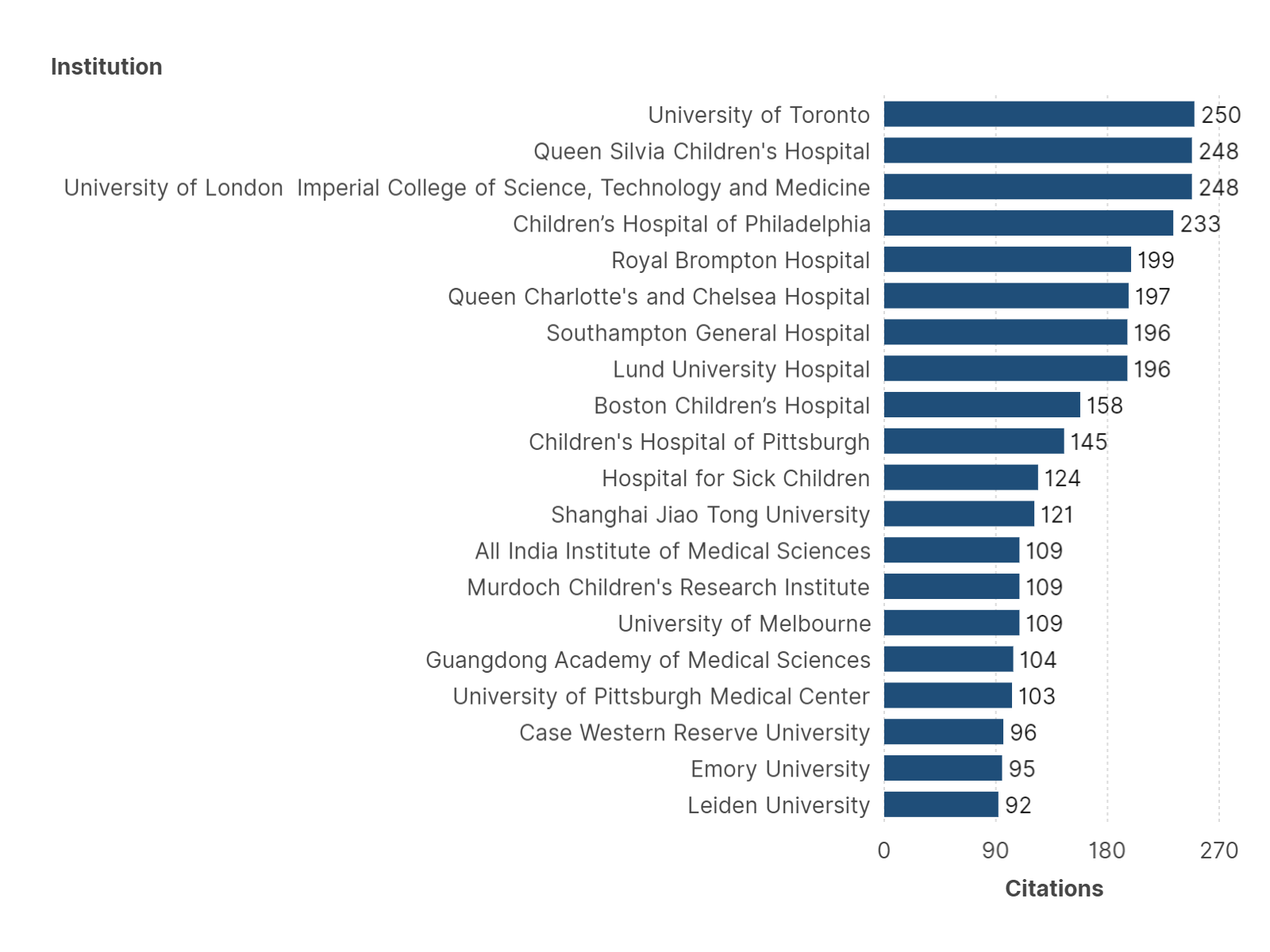


**Supplementary Fig. 6** The top 20 authors with the most citations in the field of total anomalous pulmonary venous connection


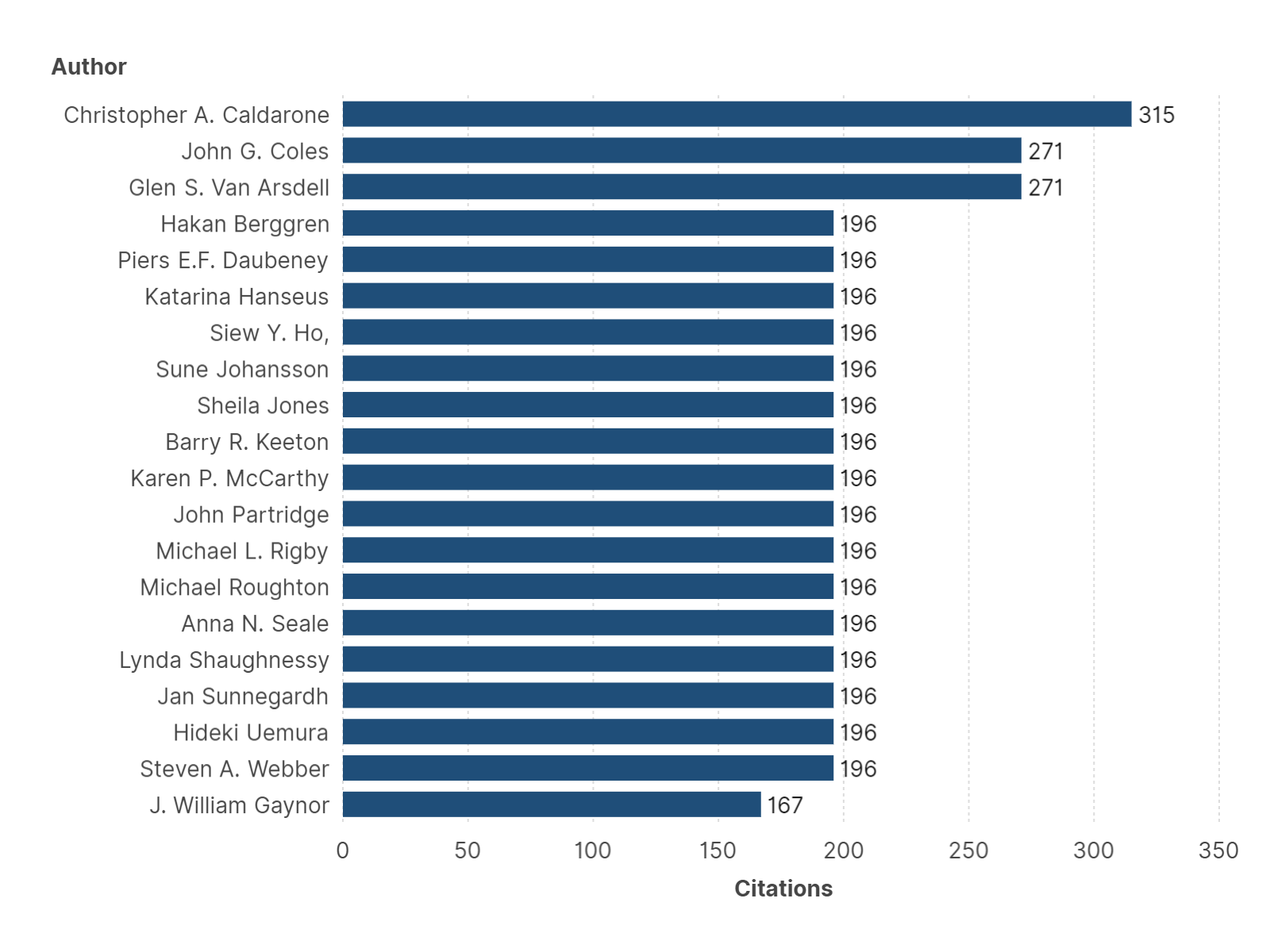


**Supplementary Fig. 7** The top 20 most frequently used keywords in the field of total anomalous pulmonary venous connection


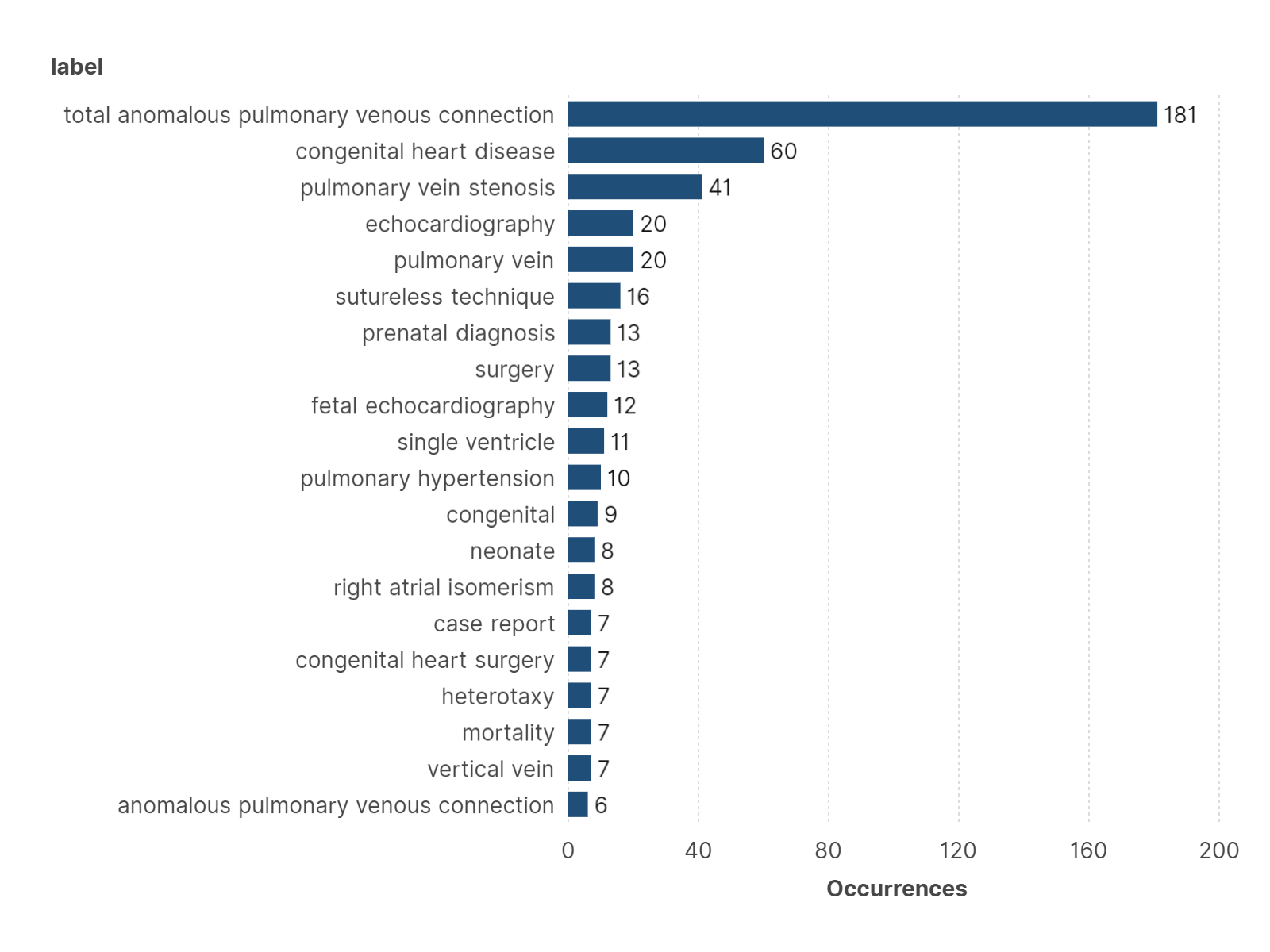

Supplement: Supplementary file 1 — Supplementary Material 1 [file 13019_2024_2787_MOESM1_ESM.docx]
